# Optimization of blasting for high-efficiency and low-damage drivage of large-section production drifts in block caving

**DOI:** 10.1038/s41598-025-32076-w

**Published:** 2025-12-13

**Authors:** Shiqian Yan, Ximing Jian, Xianglong Li, Xinglong Feng, Guangquan Li

**Affiliations:** 1 Faculty of Land Resources Engineering, Kunming University of Science and Technology, Kunming, 650093 Yunnan China; 2 Advanced Blasting Technology Engineering Research Center of Yunnan Provincial Department of Education, Kunming, 650093 Yunnan China; 3 Yunnan Diqing Non-Ferrous Metals Co., Ltd., Shangri-La, 674400 Yunnan China

**Keywords:** Block Caving, Large-section Roadway Drivage, Numerical Simulation, Reliever Hole Effect, Industrial Trial, Energy science and technology, Engineering

## Abstract

Excavating large-section production drifts in block caving mines faces critical challenges regarding surrounding rock stability and drivage efficiency. To address these issues, this study optimizes full-face blasting parameters through coupled numerical simulations and industrial verification. The investigation focuses on the cavity formation mechanism of large-diameter burn cuts and the damage evolution characteristics during full-face blasting. Simulation results indicate that a four-uncharged-hole cut design creates a symmetrical and regular free surface, effectively balancing blasting performance and economic efficiency. Furthermore, an optimal auxiliary hole spacing range is established to minimize damage to the surrounding rock. Field implementation at the -450 m level of the JAMA Mine demonstrated that the optimized scheme resulted in a well-formed roadway profile with an average half-cast factor exceeding 90%. The average advance per round stabilized at 3.31 m, representing a 6.8% improvement over the original scheme. This research establishes a high-efficiency, low-damage blasting technique for deep large-section roadway drivage, providing a valuable reference for similar engineering projects.

## Introduction

The rapid advancement of contemporary sustainable technologies is fundamentally underpinned by the large-scale and sustainable extraction and supply of mineral resources^1^. However, as shallow deposits are depleted, mining operations are increasingly moving to greater depths. In this context, the successful application of block caving, recognized as a high-efficiency and low-cost method for large-scale underground mining, becomes critically dependent on the long-term safety and stability of its undercut level infrastructure^2,3^. This infrastructure must not only withstand the dynamic and static loads imposed by the caved rock mass but is also frequently subjected to vibrations and disturbances originating from drivage and production blasting^4,5^.

**Fig. 1:**
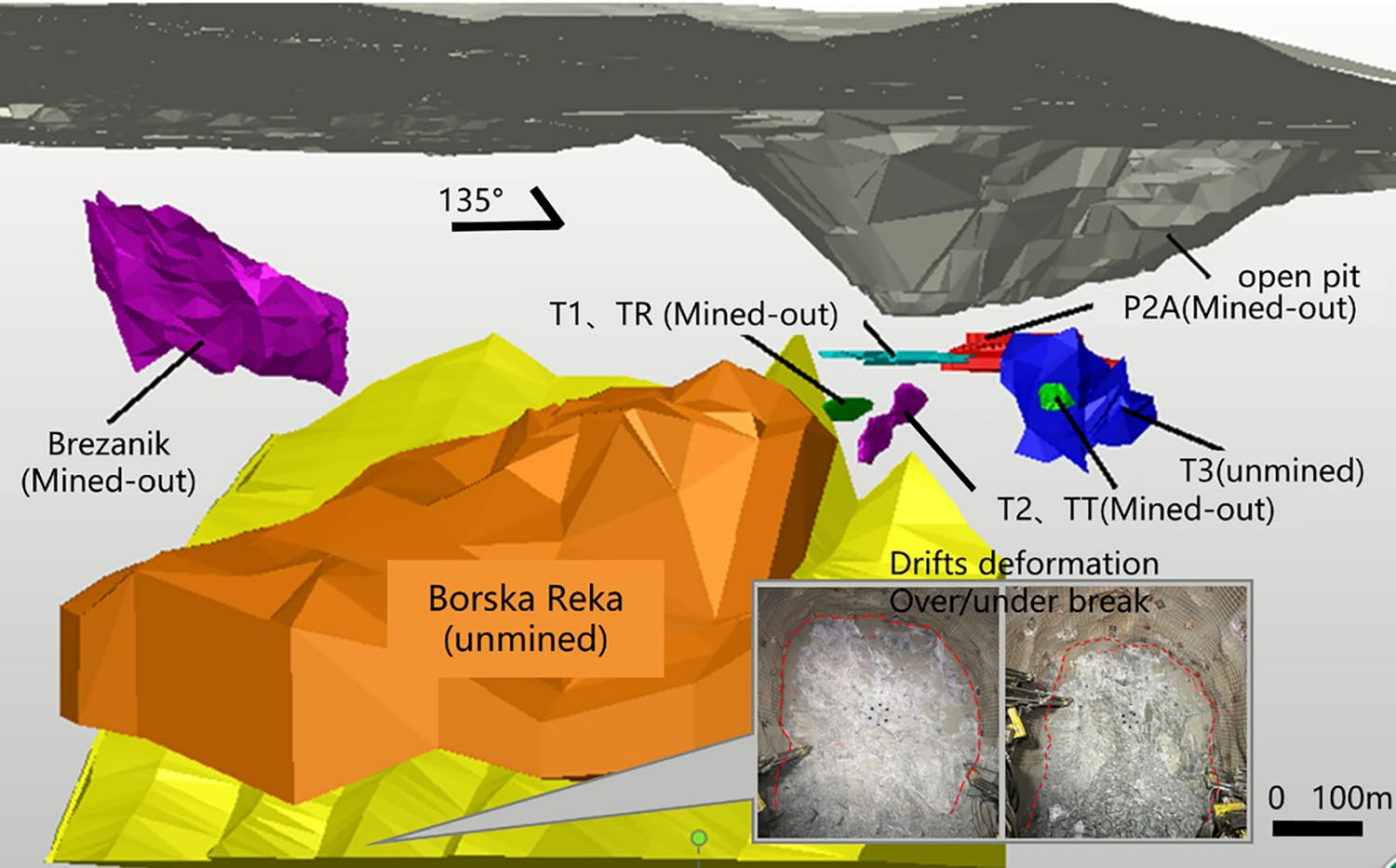
Spatial relation between orebodies and roadway instability. The 3D model depicts the mined-out zones (T1, T2, P2A) overlying the production level, creating a high-stress environment. The inset photo exhibits typical roadway spalling, indicating significant stress concentration and damage to the pillar boundaries.

The engineering background of this study is the JAMA Copper Mine, located in the Bor metallogenic belt, Serbia. The mine is developing the deep Borska Reka (BR) orebody, a giant porphyry copper deposit exceeding 1 billion tons, using an advanced intelligent block caving system. The specific study area is the undercut level at a depth of -450 m. To support a fully automated fleet, production drifts are designed with a substantial 4.5 m $\times$ 4.0 m cross-section. However, the excavation environment within the hard andesite rock mass is severe. As shown in Fig. 1, the drifts are located beneath the massive BR orebody and the mined-out high-grade zones (T1, T2, P2A, and Brezanik). Furthermore, significant voids (approximately 500,000 $m^{3}$) exist in the upper levels (-170 m to -210 m). This complex geometric relationship profoundly exacerbates stress concentrations. This results in significant sidewall spalling and poor contour formation, as highlighted in the figure (which act as direct indicators of stress-induced damage to the pillar boundaries). Consequently, mitigating excavation-induced disturbance from the very outset of the blasting drivage stage has become a critical imperative for ensuring the mine’s safe production and long-term economic viability^6,7^.

In roadway drivage using the full-face drilling and blasting method, cut blasting is the core element governing rock fragmentation efficiency and the profile of the heading, whereas perimeter blasting directly determines the final contour quality and the extent of damage to the surrounding rock^8,9^.The mechanism of the uncharged relief hole (often referred to as the ”reliever hole” or ”empty hole”) in parallel-hole cut blasting is widely recognized. Its primary functions encompass stress guiding, providing a free face, and offering compensation space for rock swell^10^. Specifically, stress waves reflect at the boundaries of these reliever holes to create tensile zones that facilitate crack propagation^11,12^. Concurrently, detonation gases are channeled through these voids, releasing energy and ejecting the fragmented rock. These combined mechanisms are decisive for efficient cavity formation and disturbance control^13^. However, optimizing the cut blasting alone is insufficient for large-section roadway drivage. Controlled blasting in the perimeter region is equally crucial, as factors including blasting parameters, charge structure, and a proper initiation sequence significantly influence the final degree of surrounding rock damage and the subsequent stress redistribution^14^.

Current research on optimizing roadway drivage focuses on two main goals. The first is enhancing the efficiency of the cut blasting. The second is controlling damage to the excavation contour^15–18^. Regarding high-efficiency excavation, burn cut blasting with large-diameter uncharged holes is widely used. The mechanism relies on the uncharged hole. It provides a free surface and compensation space for rock breaking. However, high in-situ stress creates a ’clamping effect’ on the rock. This makes rock ejection difficult. Consequently, standard cut designs often fail to achieve the expected advance per round in hard rock conditions^19,20^. Regarding low-damage control, smooth blasting techniques are essential. The primary objective is to limit the range of blast-induced cracks. This is typically achieved by optimizing the charge structure and the spacing of perimeter holes. Effective control preserves the self-supporting capacity of the surrounding rock^21–23^. However, a critical gap remains regarding the undercut level of block caving mines. These production drifts differ from standard tunnels. They are large in section and operate under complex stress superposition from overlying mined-out zones. Traditional methods often struggle to balance powerful fragmentation with the protection of pillars. Consequently, developing an integrated scheme for both high-efficiency and low-damage drivage in this specific environment is an urgent engineering challenge.

Therefore, to address the stability control demands in the drivage of large-section production drifts for block caving mines, this paper conducts comprehensive numerical simulations and field experiments covering the entire process of both cut and full-face blasting. The study aims to systematically investigate the role of reliever holes in stress wave propagation and crack extension. A primary focus is placed on optimizing the overall blasting scheme, encompassing both the cut and perimeter blasts. The ultimate goal is to establish a high-efficiency, low-damage blasting technology for large-section roadway drivage, thereby providing a theoretical basis and a technical pathway for enhancing the stability and safety of the surrounding rock in the block caving undercut level.

## Methodology

### Physical and mechanical property tests

The compressive strength tests were conducted using the RTR-1500 high-temperature, high-pressure rock triaxial testing system (GCTS). The GCTS is equipped with an axial loading system, a confining pressure loading system, a pore pressure loading system, and a high-temperature control system, making it capable of performing uniaxial compression, triaxial compression, and other related tests. The system has a maximum static loading capacity of 1500 kN, a tensile loading capacity of 820 kN, and a maximum stroke of 50 mm. It can simultaneously and independently apply, vary, and control the axial load, confining pressure, back pressure, and loading rate applied to a specimen, enabling both static and dynamic triaxial tests. It is a high-precision testing instrument widely used in engineering fields^24^.

**Fig. 2:**
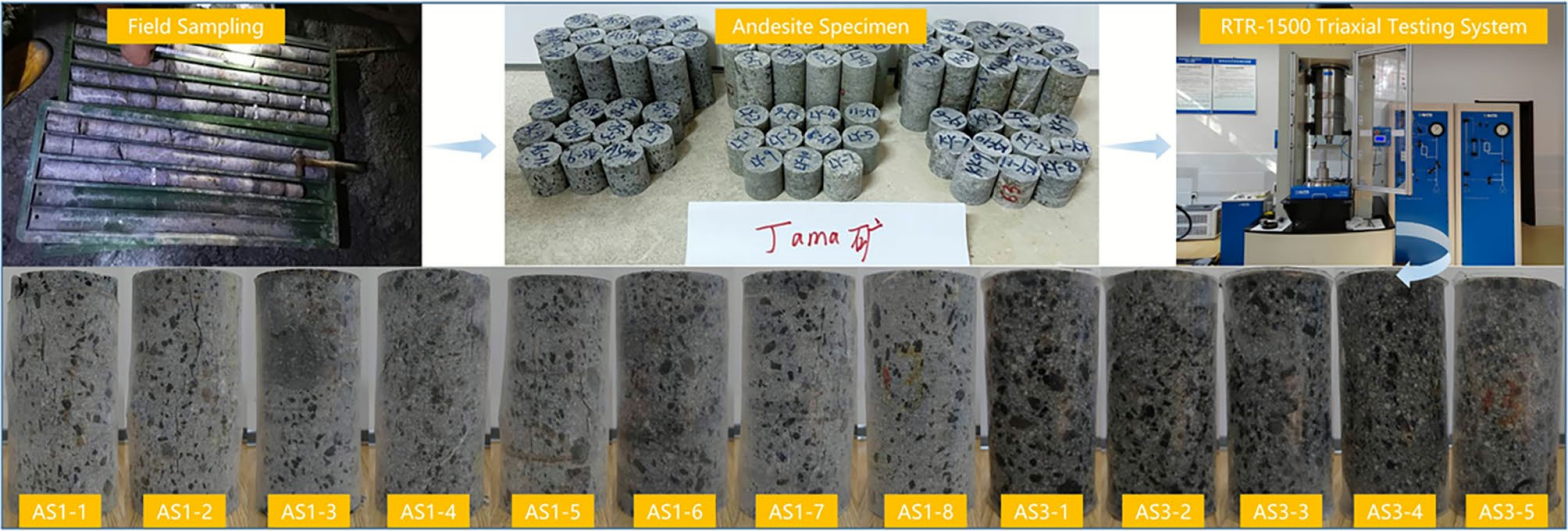
The experimental apparatus and failure modes of the andesite specimens.

Standard cylindrical specimens were prepared with a diameter of 48 mm and a height-to-diameter ratio of approximately 2:1. The mechanical properties of the andesite obtained from the tests are summarized in Table 1. Fig. 2 illustrates the experimental setup utilizing the RTR-1500 system and displays the andesite specimens before and after loading. The post-failure images clearly demonstrate the brittle fracture characteristics and shear failure planes of the rock, which validates the selection of the constitutive model parameters in the subsequent numerical simulations.

**Table 1:** Mechanical properties of andesite obtained from uniaxial and triaxial tests.

(a) Uniaxial test results							
ID	Diameter (mm)	Height (mm)	Mass (g)	Unit Weight ($\hbox {kN/m}^3$)	$\hbox {UCS}^1$ (MPa)	Young’s Modulus, $E_c$ (GPa)	Poisson’s Ratio, *v*
AS1-1	47.36	101.07	468.52	26.31	129.39	42.03	0.19
AS1-2	47.36	101.26	467.29	26.20	115.56	39.95	0.20
AS1-3	47.42	101.43	471.83	26.34	119.47	/$^2$	/$^2$
AS1-4	47.43	101.33	474.74	26.52	147.71	/$^2$	/$^2$
AS1-5	47.46	101.09	471.82	26.38	139.34	46.88	0.20
AS1-6	47.43	101.74	475.30	26.44	140.74	56.61	0.18
AS1-7	47.38	100.75	472.57	26.60	111.05	42.30	0.19
AS1-8	47.45	100.66	471.63	26.50	118.49	49.61	0.20

(b) Triaxial test results							
ID	Diameter (mm)	Height (mm)	Confining Pressure, $\sigma _3$ (MPa)	$\hbox {TCS}^3$ (MPa)	Cohesion, *c* (MPa)	Internal Friction Angle, $\varphi$ ($^{\circ }$)	
AS3-1	47.36	100.51	5	156.78			
AS3-2	47.33	102.03	10	213.32			
AS3-3	47.34	101.28	15	234.81	26.1	47.3	
AS3-4	47.34	101.23	20	272.89			
AS3-5	47.42	101.28	25	318.80			

$^1$ UCS: Uniaxial Compressive Strength.

$^2$ /: Not measured or calculated.

$^3$ TCS: Triaxial Compressive Strength.

**Fig. 3:**
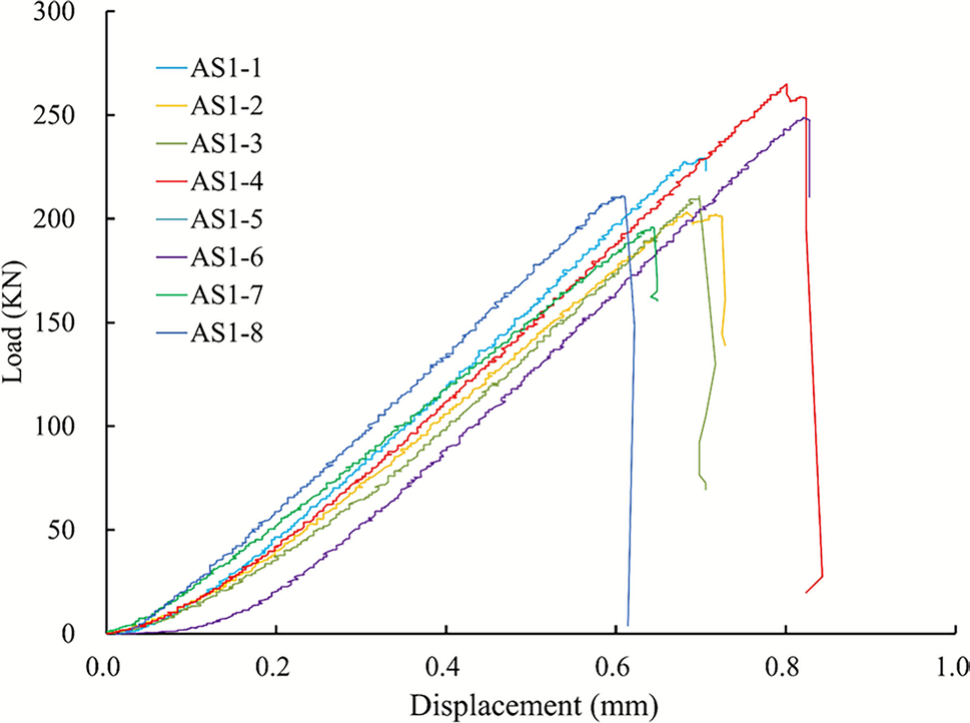
Failure modes of andesite specimens after uniaxial compressive tests.

**Fig. 4:**
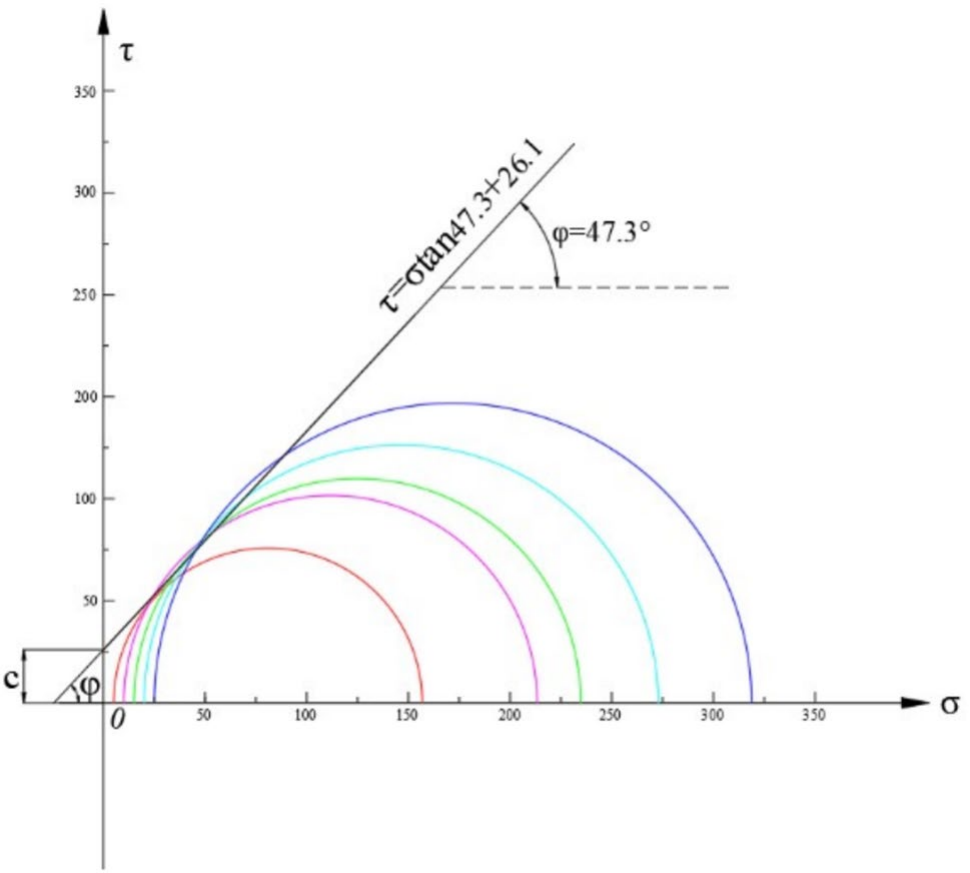
Mohr’s circles and the corresponding failure envelope for andesite.

The shear strength, characterized by the parameters of cohesion (*c*) and internal friction angle ($\varphi$), cannot be measured directly. Instead, these values are determined by combining experimental data with analytical methods. Specifically, using the results from the triaxial compression tests, Mohr circles are plotted. By constructing the common tangent line to these circles (i.e., the Mohr-Coulomb failure envelope), the cohesion and internal friction angle can be derived^25^, As illustrated in Fig. 4, a linear failure envelope is fitted tangent to the Mohr circles representing different confining pressures. This graphical method allows for the precise determination of the shear strength parameters, with the intercept representing cohesion and the slope indicating the angle of internal friction. From the uniaxial compression test, the Uniaxial Compressive Strength (*UCS*) of the andesite was determined to be 128 MPa. Based on this result and in consideration of the in-situ stress conditions, a series of triaxial compression tests were performed under confining pressures of 5 MPa, 10 MPa, 15 MPa, 20 MPa, and 25 MPa. Photos of the failed specimens from these tests are also presented in Fig. 3.

To ensure the relevance of the data, all test specimens were sourced directly from the project’s target elevation (-450 m) and were rigorously screened for defects. While the number of specimens is limited, the low degree of scatter observed in the test results (see Table 1 and Fig. 3) indicates a consistent mechanical behavior, providing confidence in their representativeness for the intact rock matrix.

### Numerical model development

With the widespread application of computer technology, numerical simulation has become an indispensable tool for addressing complex engineering and scientific problems, particularly for analyzing nonlinear dynamic processes such as blasting^26,27^. In this study, the explicit dynamic code ANSYS / LS-DYNA (Version R19.0, https://www.ansys.com/products/structures/ansys-ls-dyna) was employed to verify the proposed optimization methodology. The constitutive models and associated parameters adopted for the simulation are described in detail below.

The numerical models were established to represent the excavation process accurately. A model with dimensions of 2 m $\times$ 2 m was used for the initial cut simulation, while a larger model of 5 m $\times$ 6 m was established for the full-face excavation analysis. To ensure numerical accuracy, the overall element size was set to 1 cm. A refined mesh was employed in the immediate vicinity of the blast holes to precisely capture the high-stress gradients and fracture evolution during detonation. To simulate the far-field rock mass, non-reflecting boundary conditions were applied to the four lateral faces and the back face of the model. This technique prevents the reflection of stress waves from the model boundaries, ensuring a more realistic simulation of an infinite rock mass. Prior to the blasting simulation, an initial geostatic stress field, consistent with the in-situ stress conditions at a depth of -450 m, was applied and allowed to reach equilibrium. To control numerical oscillations inherent in explicit dynamic analyses, both hourglass control and artificial bulk viscosity were employed. An hourglass control algorithm was utilized to suppress zero-energy deformation modes in the solid elements. Concurrently, standard artificial bulk viscosity settings were applied to dampen high-frequency oscillations generated by the shockwaves, thereby maintaining the stability and accuracy of the simulation.

#### Constitutive model for rock mass

The andesite rock mass was simulated using the Riedel-Hiermaier-Thoma (RHT) model, a constitutive model designed for brittle materials under dynamic loading. The model parameters, presented in Table 2, were calibrated based on the static laboratory test results obtained in this study and the dynamic mechanical properties determined in our previous research^28,29^.

**Table 2:** RHT constitutive model parameters for andesite.

Parameter	Symbol	**Value**
**Basic & Equation of State (EOS) Parameters**		
Initial Density ($\hbox {kg/m}^3$)	$\rho _0$	2690
Solid Density ($\hbox {kg/m}^3$)	$\rho _e$	2715
Elastic-Limit Pressure (GPa)	$p_e$	0.02
Lock-up Pressure (MPa)	$p_I$	42.5
Porosity Exponent	*n*	3
Initial Porosity	$\alpha$	0.025
Shear Modulus (GPa)	*G*	46.23
EOS Parameter T1 (GPa)	$T_1$	24.85
EOS Parameter T2 (GPa)	$T_2$	0
**Strength Parameters**		
Uniaxial Compressive Strength (MPa)	$f_c$	127
Tensile to Compressive Strength Ratio	$f_t/f_c$	0.1
Shear to Compressive Strength Ratio	$f_s/f_c$	0.3
Intact Failure Surface Constant	*A*	0.88
Intact Failure Surface Exponent	*N*	0.65
**Strain Rate Parameters**		
Failure Surface Constant for Strain Rate	*B*	1.62
Failure Surface Exponent for Strain Rate	*M*	0.61
Strain Rate Parameter	$B_0$	0.9
Strain Rate Parameter	$B_1$	0.9
Strain Rate Parameter	$B_Q$	0.0105
Strain Rate Constant	$Q_{2,0}$	0.7
**Third Invariant & Residual Strength Parameters**		
Compressive Meridian Ratio	$R_C$	0.53
Tensile Meridian Ratio	$R_t$	0.7
GDR Model Parameter	$R_G$	2
Residual Strength Parameter	$F_G$	0.13
**Damage Parameters**		
Damage Constant	$D_1$	0.04
Damage Exponent	$D_2$	1
Damage Evolution Parameter	$\delta$	0.02

The RHT model captures the pressure-dependent behavior of the rock through two primary stages. It begins with an elastic phase, where the initial porosity of the rock decreases under increasing compressive pressure until crushing occurs. This is followed by a plastic phase, where the crushed material undergoes further compaction at higher pressures. The model’s pressure-volume relationship is governed by a polynomial equation of state suitable for porous materials.

1 $$\begin{aligned} & p=\left\{ \begin{array}{l} A_{1} \bar{\mu }+A_{2} \bar{\mu }^{2}+A_{3} \bar{\mu }^{3}+\left( B_{0}+B_{1} \bar{\mu }\right) \rho _{0} E_{0}, \bar{\mu }>0 \\ T_{1} \bar{\mu }+T_{2} \bar{\mu }^{2}+B_{0} \rho _{0} E_{0}, \bar{\mu }<0 \end{array}\right. \end{aligned}$$

2 $$\begin{aligned} & \bar{\mu } = \frac{\rho _{e} \alpha (p)}{\rho _{0}} - 1 \end{aligned}$$

3 $$\begin{aligned} & a(p)=1+\left( a_{e}-1\right) \left[ \frac{p_{I}-p}{p_{I}-p_{e}}\right] ^{n} \end{aligned}$$

For fully compacted material, Equation (1) simplifies to:

4 $$\begin{aligned} & p=\left\{ \begin{array}{l} A_{1} \mu +A_{2} \mu ^{2}+A_{3} \mu ^{3}+\left( B_{0}+B_{1} \mu \right) \rho _{0} E_{0}, \mu >0 \\ T_{1} \mu +T_{2} \mu ^{2}+B_{0} \rho _{0} E_{0}, \mu <0 \end{array}\right. \end{aligned}$$

5 $$\begin{aligned} & \mu = \frac{\rho }{\rho _{0}} - 1 \end{aligned}$$

where $E_{0}$ is the initial internal energy of the material; $\rho$ is the current density of the material during compression; $\rho _{0}$ is the initial density of the material; $\rho _{e}$ is the density of the material at the elastic limit pressure; $\alpha _{0}$ is the initial porosity of the material; $\alpha _{e}$ is the porosity at the onset of pore collapse; *p* is the pressure; $p_{e}$ is the pressure at which pores begin to crush (i.e., the elastic-limit pressure); $p_{0}$is the pressure at which the material is fully compacted (i.e., the lock-up pressure); $A_{1}$, $A_{2}$, and $A_{3}$ are the coefficients of the Hugoniot polynomial; $T_{1}$, $T_{2}$, $B_{0}$, and $B_{1}$ are parameters for the equation of state;*n* is the porosity exponent.

#### Equation of state for explosives

The pressure generated by the detonation products of the explosive is described by the Jones-Wilkins-Lee (JWL) equation of state^30^, which has the following form:

6 $$\begin{aligned} p_{j}=A_{j}\left[ 1-\frac{\omega }{R_{1} V}\right] E_{1}^{-R_{1} V}+B_{j}\left[ 1-\frac{\omega }{R_{2} V}\right] E_{1}^{-R_{2} V}+\frac{\omega E_{e}}{V} \end{aligned}$$

Where $p_{j}$ is the pressure of the detonation products, *V* is the relative volume, and $E_{c}$ is the specific internal energy. $A_{j}$, $B_{j}$, $R_{1}$, $R_{2}$, and $\omega$ are material constants specific to the explosive. The parameters for the emulsion explosive used in this study are listed in Table 3. These values are based on the work of Li et al.^31^ and include the initial density ($\rho _{j}$) and detonation velocity ($v_{d}$).

**Table 3:** JWL parameters for 2# rock emulsion explosive.

Parameter	Symbol	Value
Initial Density ($\hbox {g/cm}^3$)	$\rho _j$	1.25
Detonation Velocity (m/s)	$v_d$	3200
C-J Detonation Pressure (GPa)	$p_j$	9.53
JWL Constant (GPa)	$A_j$	276.2
JWL Constant (GPa)	$B_j$	8.44
JWL Constant (dimensionless)	$R_1$	5.2
JWL Constant (dimensionless)	$R_2$	2.1
JWL Constant (dimensionless)	$\omega$	0.57
Initial Specific Internal Energy per Unit Volume (GPa)	$E_1$	3.87

#### Material model for air

In the numerical simulation, the behavior of air was captured using the *MAT-NULL material model available in LS-DYNA, which allows for the definition of material behavior primarily through an equation of state^32^. The air pressure, $p_{A}$, is governed by the linear polynomial EOS, expressed as:

7 $$\begin{aligned} P_{A}=C_{0}+C_{1}+C_{2} \lambda ^{2}+C_{3} \lambda ^{3}+\left( C_{4}+C_{5} \lambda +C_{6} \lambda ^{2}\right) E_{2} \end{aligned}$$

where $E_{2}$ is the internal energy per unit volume and $\lambda$ is the dynamic viscosity coefficient. Based on the ideal gas assumption for air, the only non-zero EOS coefficient is $C_{4}$, which was set to 0.401, while $C_{1}$, $C_{2}$, $C_{3}$, $C_{5}$, and $C_{6}$ were defined as zero.

### Blasting parameter design strategy

To address persistent issues such as low advance rates and significant overbreak in current ore-pass roadway drivage, this study focuses on the optimization of the cut and smooth blasting phases. The primary objectives are to enhance the excavation efficiency, improve the contour quality of the final roadway profile, and thereby reduce subsequent ground support costs.

#### Theoretical calculation of the cut pattern

**Fig. 5:**
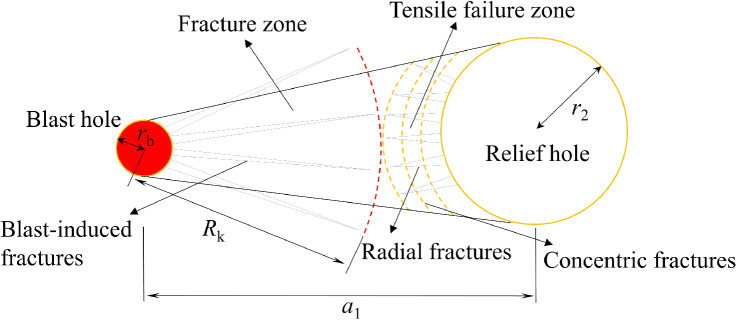
Positional relationship between a charged hole and a reliever hole.

The spacing between charged holes and adjacent uncharged relief holes is a critical parameter that governs the effectiveness of cavity formation during cut blasting (Fig. 5). In deep-hole excavation, shockwaves generated by the detonation of a charged hole induce a high-stress concentration around the perimeter of a nearby reliever hole. This phenomenon facilitates the generation of reflected tensile waves, which are highly effective in fracturing the rock mass. This mechanism is essential for creating an initial cavity of adequate size and volume, a prerequisite for the success of the main blasting round^33^.

Therefore, the design of the spacing, must ensure thorough rock fragmentation between the holes. Simultaneously, practical constraints such as drilling accuracy must be considered to prevent hole intersection. The design adheres to the following principles:

First, the reliever hole must be located within the fracture zone radius ($R_{k}$) generated by the charged hole. This establishes the upper bound for the spacing: $a_{1} < R_{k}$.

Conversely, the spacing cannot be too small, as this could lead to excessive pulverization of the rock, potentially choking the cavity and obstructing the ejection of the muckpile. Thus, the spacing a must also satisfy a lower bound, defined by Eq. (8):

8 $$\begin{aligned} \frac{a_{1}\left( r_{b}+r_{2}\right) +\left( r_{b}^{2}+r_{2}^{2}\right) }{a_{1}\left( r_{b}+r_{2}\right) -\left( r_{b}^{2}+r_{2}^{2}\right) }>m \end{aligned}$$

where: $r_{b}$ is the radius of the charged hole (m); $r_{2}$ is the radius of the large reliever hole (m); *m* is the initial compensation coefficient, typically ranging from 1.0 to 1.4. Based on site-specific parameters and the principles above, the calculated optimal spacing ($a_{1}$) between the charged and reliever holes was determined to be in the range of 30 to 50 cm.

#### Theoretical calculation of perimeter hole spacing and minimum burden

The fracturing mechanism between perimeter holes is a two-stage process. Upon detonation, the blast hole wall is subjected to the initial impact of the detonation wave, followed by the sustained pressure of the expanding detonation gases. This action generates a radial stress field that attenuates with increasing distance from the hole.

It is widely accepted that the formation of a continuous fracture between adjacent holes is not primarily caused by the superposition of stress waves. Rather, the mechanism involves two distinct phases^34^: 1. Initial Fracturing: The stress wave induced by the detonation in each hole first creates a network of initial, small-scale fractures in the immediate vicinity of the blast hole wall. 2. Fracture Propagation and Coalescence: Subsequently, the quasi-static pressure from the detonation gases penetrates these initial fractures. This sustained pressure drives their propagation until they coalesce with fractures extending from the adjacent hole, forming a continuous fracture plane.

Therefore, the spacing of the perimeter holes is determined using a quasi-static mechanical model that accounts for this gas pressure-driven fracture propagation, as follows:

9 $$\begin{aligned} a_{2}=2\left( R_{k}+r_{b}\left( \frac{p_{b}}{\sigma _{t d}}\right) \right) \end{aligned}$$

where: $a_{2}$ is the spacing between perimeter holes; $p_{b}$ is the quasi-static pressure exerted by the detonation gases within the blast hole. $\sigma _{td}$ is the dynamic tensile strength of the rock. By substituting the physical and mechanical rock properties obtained from laboratory tests, the required spacing was calculated to be $a_{2}$ = 0.59 m. Based on this result, a practical spacing of 60 cm was adopted for the design.

For smooth blasting operations, the minimum burden (*W*) is directly related to the spacing of the perimeter holes ($a_{2}$). This relationship is defined by the spacing-to-burden ratio ($k = a/W$), which is a key design parameter. For effective contour control, this ratio is typically kept within the range of 0.8 to 1.0. Given the previously adopted spacing of $a_{2}$ = 60 cm, the corresponding range for the burden was calculated. The final design value for the minimum burden *W* was determined to be in the range of 60 to 78 cm.

#### Establishment of simulation schemes

The burn cut, as the crucial first step in tunnel drivage blasting, directly dictates the advance per round and the overall blasting efficiency. Therefore, optimizing the burn cut design is of paramount importance for improving blasting performance^35^. In this study, based on theoretical calculations and practical engineering conditions, the spacing between the charged holes and the empty reliever holes in the cut area was set to a range of 30–50 cm. Accordingly, four distinct cut designs were developed.

**Fig. 6:**
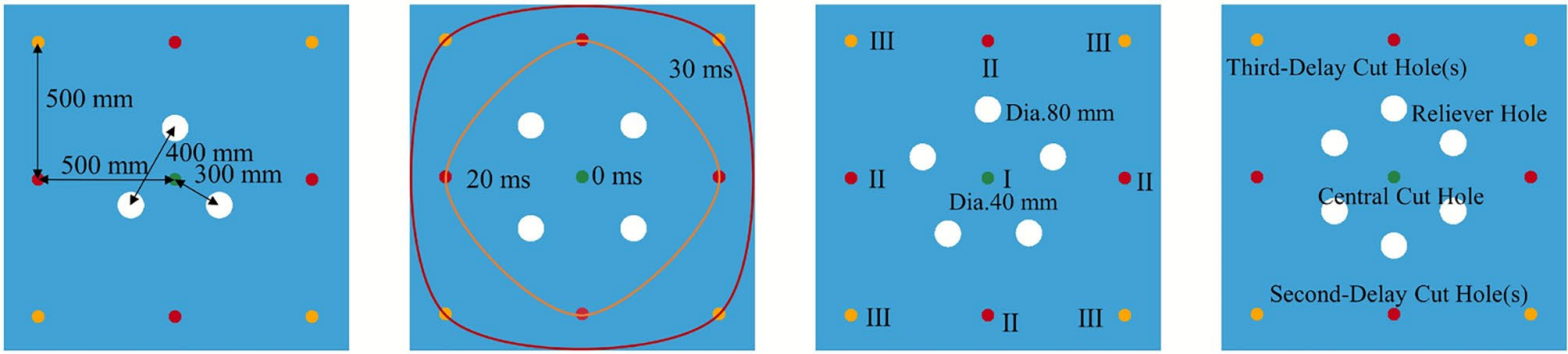
Positional relationship between a charged hole and a reliever hole.

The LS-DYNA software was employed to systematically compare the blasting effects. To determine the optimal cut structure, four distinct simulation schemes were designed based on the number of reliever holes. The specific layouts are strictly defined as follows, corresponding to the diagrams in Fig. 6: Scheme A features a 3-hole cut design where reliever holes are arranged in an equilateral triangle around the central charged hole, serving as the baseline for void volume. Scheme B employs a 4-hole cut design with reliever holes arranged in a symmetrical diamond pattern to test the effect of geometric symmetry on stress distribution. Scheme C utilizes a 5-hole cut design where reliever holes form a pentagonal shape to increase the available compensation space. Finally, Scheme D adopts a 6-hole cut design with reliever holes arranged in a hexagonal pattern to maximize the initial void volume. In all four schemes, the spacing between the central charged hole and the adjacent reliever holes was maintained constant at 300 mm. This ensures that the number of reliever holes is the sole independent variable. The objective is to evaluate the trade-off between the quality of the formed cavity and the drilling workload.

## Results: numerical simulation and analysis

### Numerical simulation of the burn cut

**Fig. 7:**
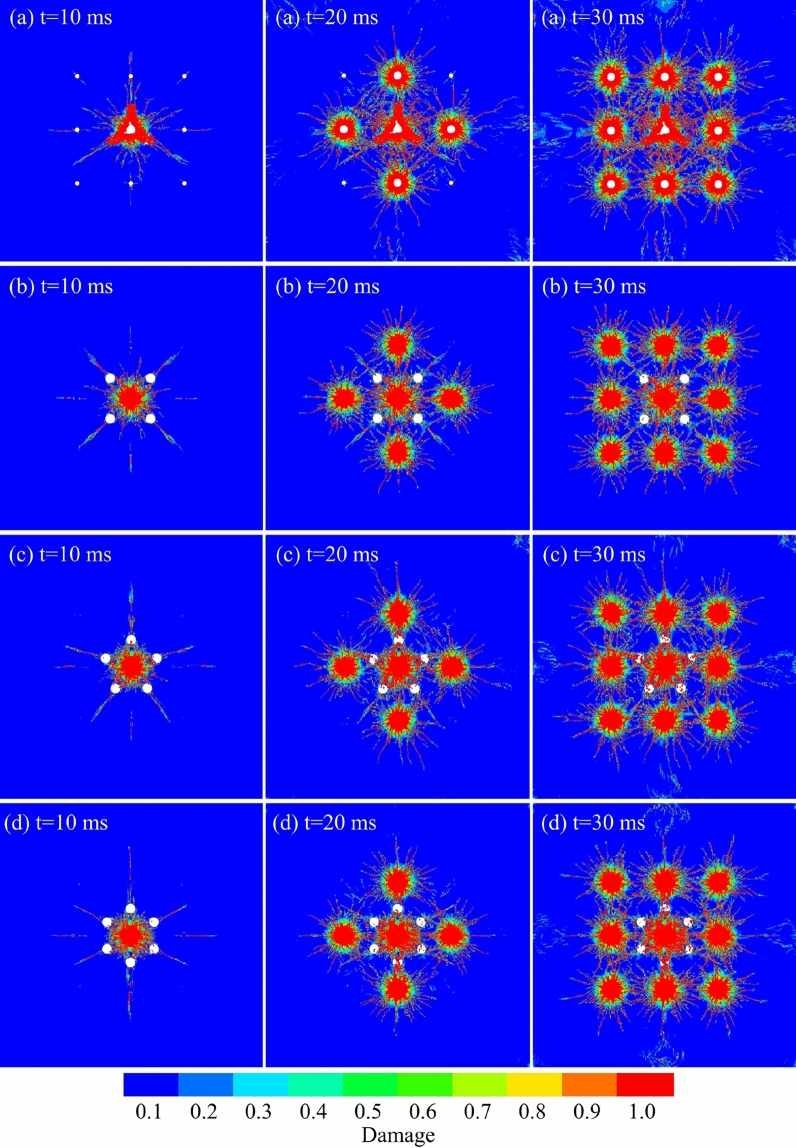
Comparison of damage zone evolution for burn cut designs with different numbers of reliever holes. (**a**) The three-reliever-hole burn cut. (**b**) An isolated four-reliever-hole burn cut. (**c**) An isolated five-reliever-hole burn cut. (**d**) An isolated six-reliever-hole burn cut.

**Fig. 8:**
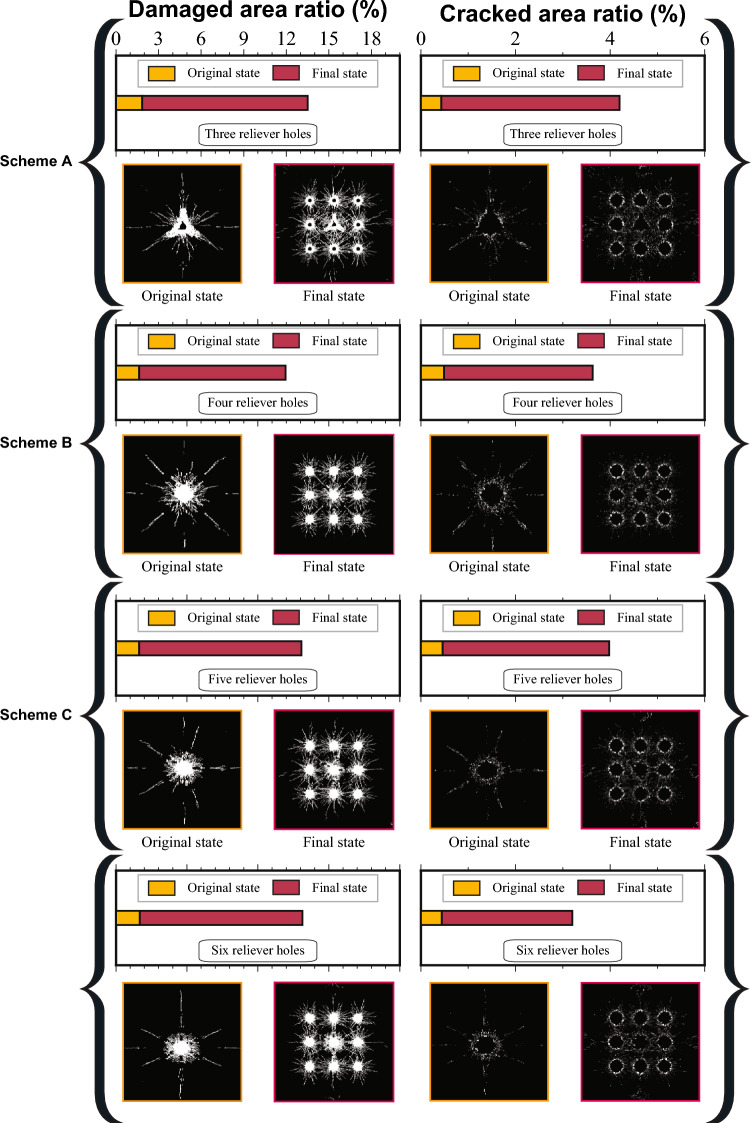
Quantitative comparison of damaged and cracked area evolution characteristics for burn cut schemes with varying numbers of reliever holes.

Throughout the simulation, the dynamic propagation of the damaged and cracked area under a sequential delay detonation sequence was tracked. The evolution of the percentage of these zones at critical time steps for each design is presented in Fig. 7. To quantify the blasting effect, a MATLAB image processing algorithm was utilized to analyze the damage distribution. The simulation results were segmented based on the RGB color values of the damage contours. Red areas were identified as the damaged area (Damage variable D$\ge$0.75), while green areas represented the cracked area (0.2$\le$ D<0.75). The percentage of each area was calculated by the ratio of recognized pixels to the total area. As illustrated in Fig. 8, in Scheme A, following the detonation of the central hole (t=10 ms), the initial percentages of the damaged and cracked areas were 1.85% and 0.43%, respectively. As the subsequent delay charges detonated, these zones expanded rapidly, reaching 13.51% and 4.20% by t=30 ms. For Scheme B, the initial percentages at t=10 ms were 1.64% and 0.49%, which grew to 11.95% and 3.65% by the end of the blast. Scheme C exhibited damaged and cracked areas of 1.64% and 0.46% at t=10 ms, rising to 13.07% and 3.98% at t=30 ms. Finally, Scheme D showed initial percentages of 1.68% and 0.44%, evolving to 13.14% and 3.82% by t=30 ms.

A comprehensive analysis reveals that the effectiveness of a burn cut is not solely determined by the final volume of fractured rock, even though the 4-hole design resulted in a slightly smaller total damage zone. The crucial factors are the quality and efficiency of the initial free face creation. The 4-hole design creates a geometrically symmetrical initial cavity, which provides a uniform, four-directional relief space and stress-release conditions for the subsequent blasting of the easer holes. This, in turn, facilitates effective control of the tunnel contour and reduces the generation of oversize rock.

In contrast, while increasing the number of holes to five or six yields a marginal increase in the damage zone, it exhibits significantly diminishing returns. Meanwhile, the associated drilling costs and construction time increase substantially. Therefore, the large-diameter four-hole burn cut is identified as the optimal design, balancing blasting performance with cost-effectiveness. This optimal cut design was subsequently incorporated into the full-face blasting model to optimize the remaining blast parameters.

### Numerical simulation of full-face blasting

**Fig. 9:**
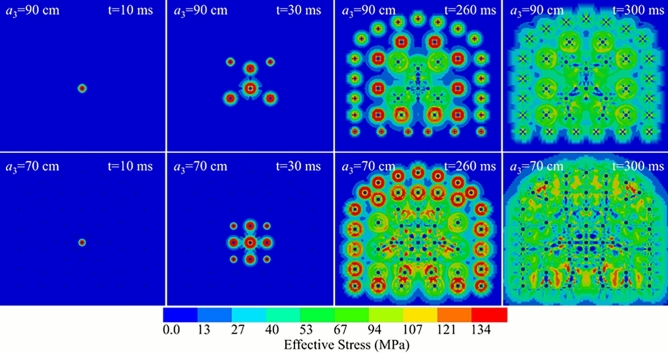
Simulation of the stress evolution process during blasting for roadway excavation.

A numerical model for full-face blasting was developed based on the experimental blast hole layout. The model represents a tunnel cross-section of 4.5 m $\times$ 4.0 m. The charged holes had a diameter of 40 mm, while the reliever holes had a diameter of 80 mm. The analysis features a four-reliever-hole burn cut design, with uncoupled charging employed for the perimeter holes to create a smooth blasting layer of 70 cm. The spacing of the auxiliary holes, denoted as a, was varied across five levels: 70 cm, 75 cm, 80 cm, 85 cm, and 90 cm (this range was selected as it brackets the theoretically calculated optimal spacing). Simulations were conducted for all five spacing levels (70–90 cm). The results indicated that the variation in stress evolution and damage patterns across these intervals is continuous and gradual. The intermediate cases (75, 80, and 85 cm) exhibited characteristics consistent with the trend defined by the boundaries. Therefore, to succinctly illustrate the mechanism of stress wave interaction and avoid redundancy, the two boundary conditions, $a_{3}$ = 70 cm (strongest superposition) and $a_{3}$ = 90 cm (weakest superposition), were selected as representative models for detailed comparative analysis, as shown in Fig 9. Based on the propagation and interaction characteristics of the blasting stress waves at different stages, several representative time points were selected for analysis.

In the model with an auxiliary hole spacing of 90 cm, the cut holes were arranged in a triangular pattern and detonated in two delays. For the a = 70 cm model, a four-reliever-hole cluster burn cut was used, detonated in three delays. An equal delay interval of 10 ms was set between stages.

After the central cut hole detonates (t=0 ms, reference time), the shockwave propagates outwards, forming an initial compressive stress failure zone. This creates additional free faces and relief volume for subsequent detonations. By the time all cut holes have detonated (t=30 ms), the stress waves from each hole interact. The superposition of these waves and the resulting stress concentrations cause the rock between adjacent holes to fracture and connect. Simultaneously, stress concentration around the reliever holes promotes further fracturing, forming a larger initial cavity that provides a significant free face and relief volume for the upcoming blasts. The detonation of the auxiliary holes then results in complete fragmentation of that region. Following the detonation of the perimeter holes (t=200 ms), their stress waves superpose and interact with the effects from the auxiliary holes, creating a smooth wall profile and outlining the basic contour of the roadway. In the final stage (t=300 ms), the stress waves gradually dissipate, and the final blasted face is formed.

Rock is considered severely damaged when the cumulative damage variable exceeds 0.75, indicating major structural failure^36^. A damage value between 0.2 and 0.75 signifies that the rock has been damaged and the structure is partially compromised. A value between 0 and 0.2 suggests that while minor damage may have occurred, the fundamental structure of the rock remains largely intact.

**Fig. 10:**
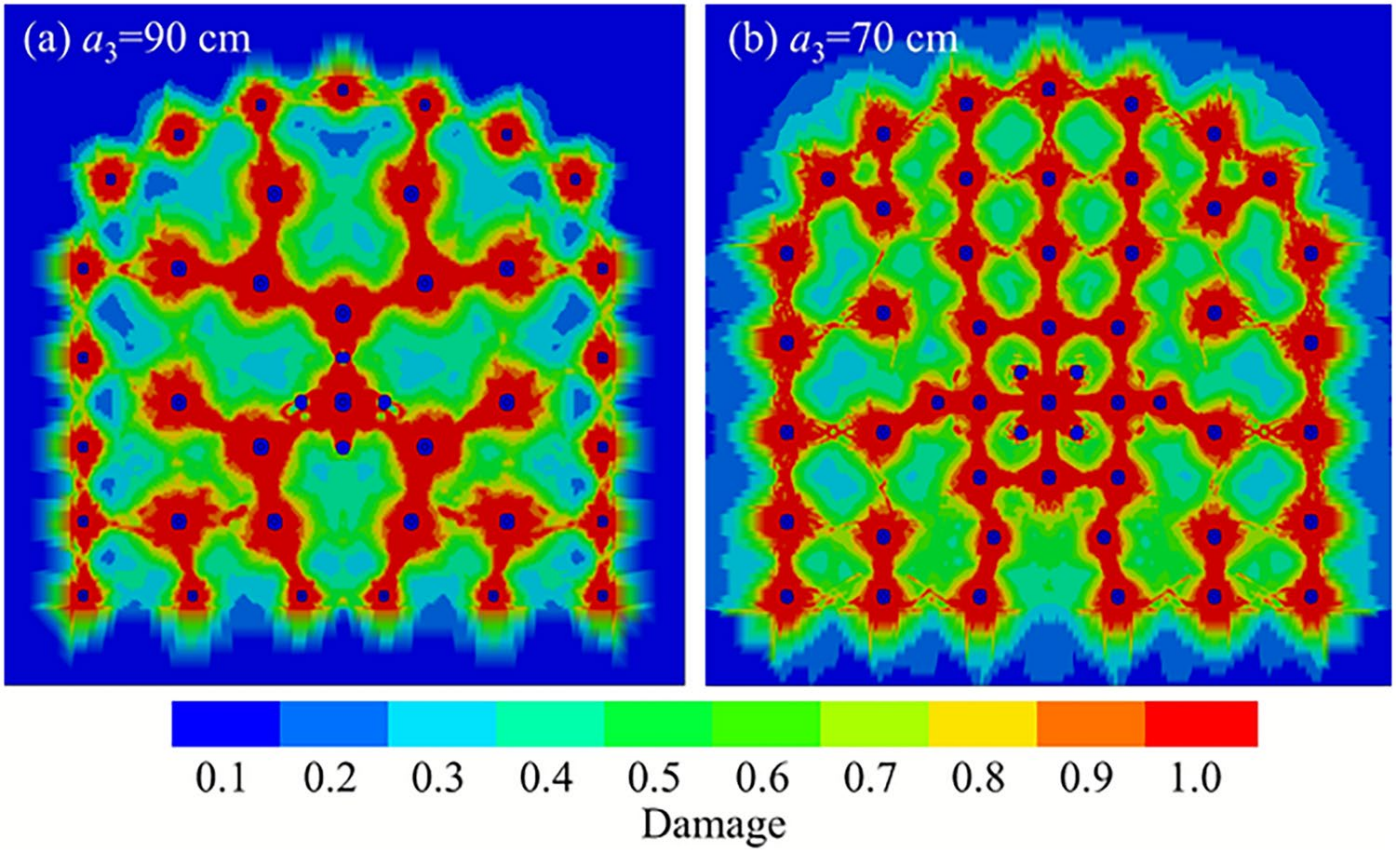
Cumulative damage contours from a full-face blasting simulation of roadway excavation.

As shown in Fig. 10, both full-face blasting designs achieve a relatively regular and smooth final contour. In both cases, the fractures between the top perimeter holes are well-connected, indicating an effective smooth blasting outcome. However, for the a = 70 cm case, the cumulative rock damage across the entire cross-section is more severe. The fracture connectivity between perimeter holes is excellent, and the superposition of stress between auxiliary holes leads to effective tensile and shear failure of the intervening rock. In contrast, for the $a_{3}$ = 90 cm case, while fracture connection between some auxiliary holes is good, the outcome in the upper part of the blasted face is less ideal. The larger spacing results in a weaker stress wave superposition effect, and the rock between these holes sustains less damage. This can lead to the formation of oversized fragments (boulders), complicating subsequent mucking operations. Meanwhile, the four-reliever-hole burn cut in this configuration shows complete interconnection between holes, successfully creating a large free face and ample relief volume.Therefore, based on these simulation results, an auxiliary hole spacing in the range of 70 cm to 90 cm is recommended for full-face blasting in roadway excavation to achieve optimal performance.

## Field validation

### Field implementation of the optimized blasting scheme

**Fig. 11:**
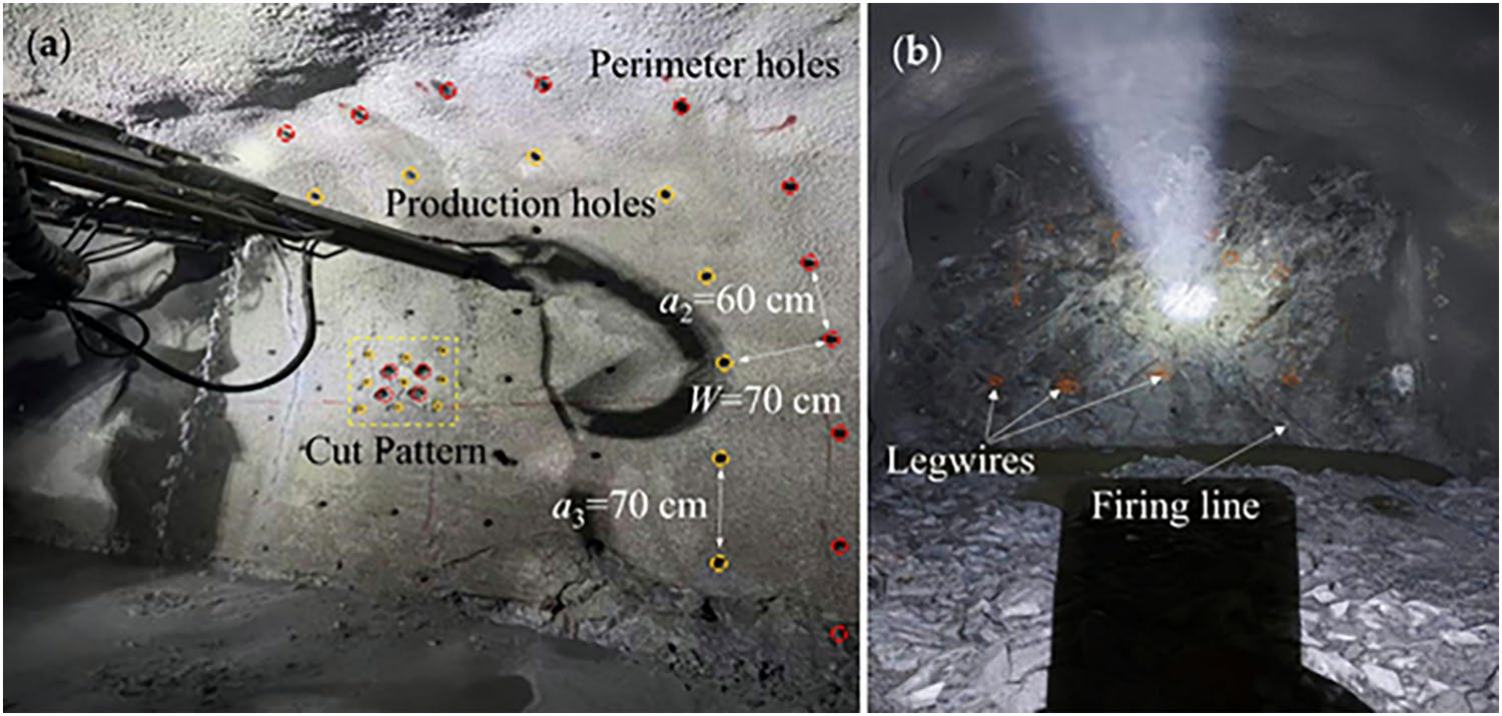
On-site operations for the field trial. (**a**) Drilling process with a jumbo drill. (**b**) Charging pattern diagram.

Industrial-scale field trials were conducted at the –450 m level of the JAMA Mine to validate the optimized full-face blasting scheme. This scheme incorporates the large-diameter four-hole burn cut developed in the preceding numerical study. The performance of the optimized scheme was comprehensively evaluated and systematically compared with the mine’s original design based on multiple metrics, including cyclic advance final contour quality, and techno-economic indicators. As shown in Fig. 11, the trials were implemented in the drifting faces of the west vein-following drift and cross-cut tunnel on the –450 m level. The surrounding rock mass in this section is andesite, characterized by good integrity with undeveloped joints and fractures. The Protodyakonov hardness coefficient (*f*) of the rock is approximately 8–10, classifying it as a hard rock mass, which is fundamentally consistent with the rock mechanical parameters used in the numerical simulations.

### Comparative analysis of blasting outcomes

**Fig. 12:**
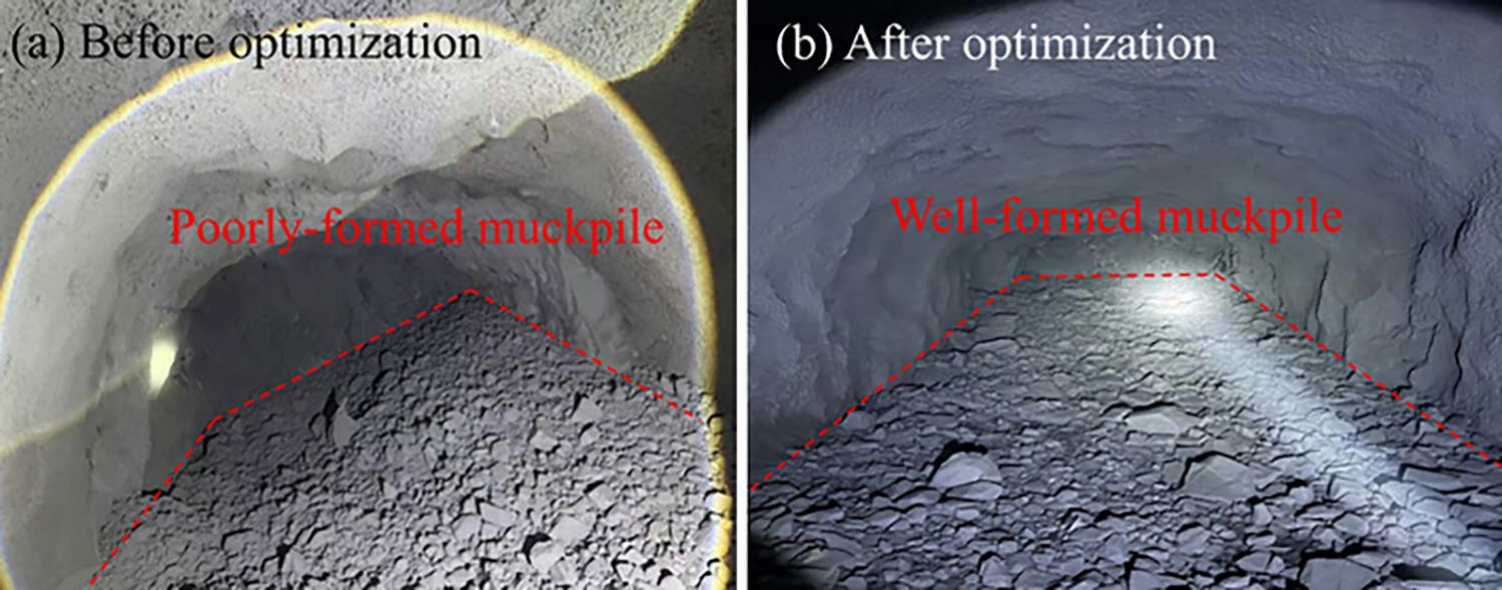
Muckpile shape before and after optimization.

The muckpile is a key indicator for evaluating the effectiveness of the burn cut and the degree of rock fragmentation. As shown in Fig. 12, the muckpile from the optimized scheme exhibited several desirable characteristics. The blasted rock was centrally and neatly piled near the tunnel centerline without excessive scattering or accumulation in corners of the face, which greatly facilitated the subsequent mucking operations. The rock fragments in the muckpile were uniform in size, with no significant boulders, indicating thorough fragmentation and high energy utilization efficiency. The front of the muckpile was flush, and no bootlegs (residual sections of charged drill holes) were observed, demonstrating that the large-diameter four-reliever-hole burn cut provided sufficient relief and compensation space for the subsequent blast holes, enabling efficient blasting of the entire tunnel face.

**Fig. 13:**
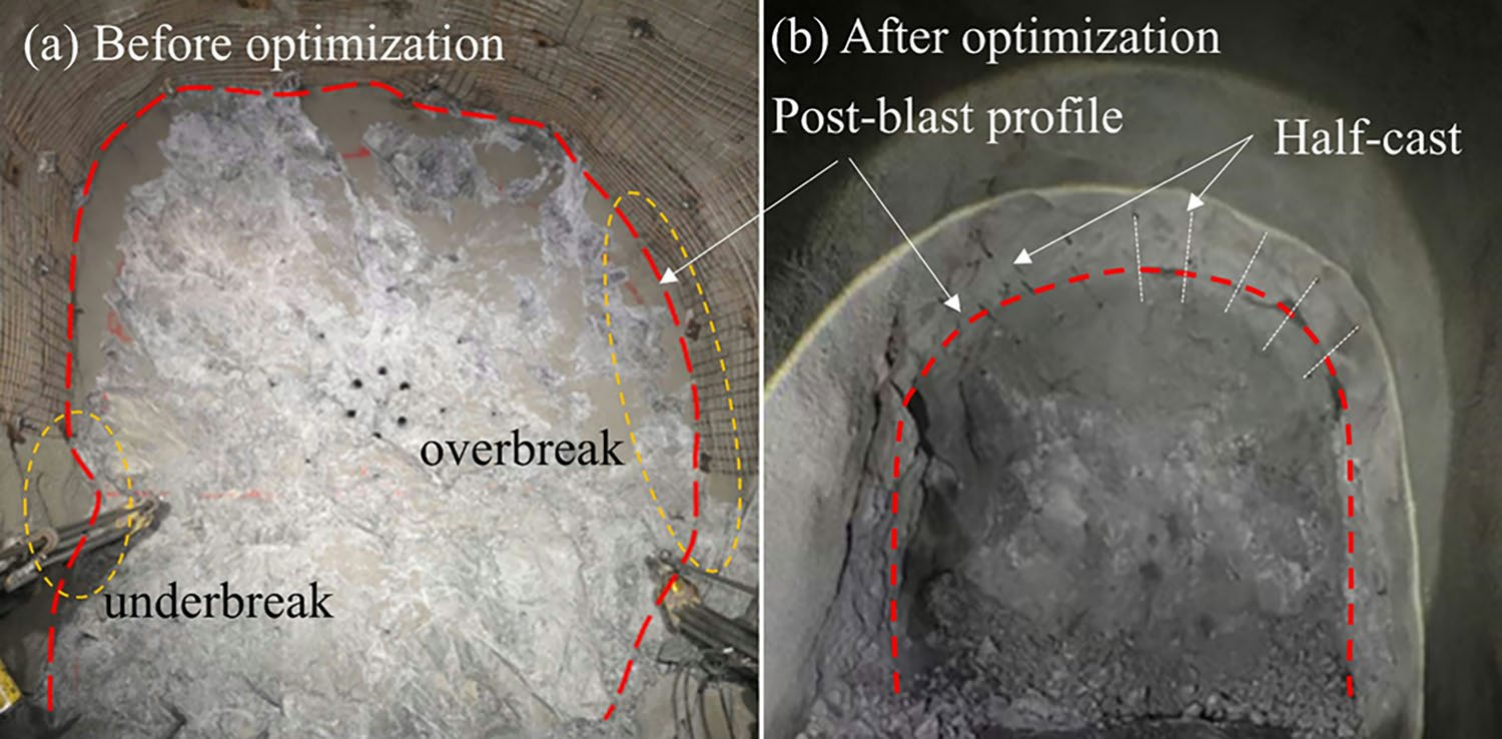
Tunnel contour before and after optimization.

The final excavated contour of the tunnel is the primary criterion for assessing the effectiveness of smooth blasting. As seen in the blasting results in Fig. 13, the tunnel profile achieved with the optimized scheme was of excellent quality. The contour line was smooth and regular, closely matching the designed profile, with remarkable control over overbreak and underbreak. The surrounding rock on the sidewalls and roof maintained high integrity, showing no significant new blast-induced fractures or rock slabbing, which effectively minimized disturbance to the rock mass. Continuous and distinct half-cast marks were visible on the tunnel walls. On-site sampling statistics revealed an average half-cast factor exceeding 90%. This serves as direct and compelling evidence of an ideal smooth blasting outcome, indicating that the blasting energy was precisely confined to the excavation boundary, thereby maximally protecting the remaining rock mass.

### Quantitative analysis of cyclic advance

To quantitatively assess the benefits of the optimized scheme, production data from all working faces applying the new design were tracked and compiled over a three-month period (June to August). The aggregated results are presented in Table 4.

**Table 4:** Statistical summary of average advance per round using the optimized scheme (June–August).

Month	Total Advance (m)	Total Cycles	Average Advance Per Round (m/round)
June	80.26	25	3.21
July	254.49	77	3.31
August	231.22	69	3.35
**Total/Overall Average**	565.97	171	3.31

Table 4 reveals a significant increase in the average advance per round. Over a three-month trial period covering multiple working faces and a total of 171 blasting cycles, the average advance per round stabilized at 3.31 m. Compared to the mine’s original average of approximately 3.1 m per cycle, this represents an improvement of approximately 6.8%. This enhancement is both significant and consistent, directly demonstrating the substantial advantage of the optimized scheme in improving excavation efficiency.

The statistical data encompassed both cross-cut and vein-following drifts on the –450 m level. The stability of the data across these varied conditions confirms the scheme’s wide applicability and reliability. The ability to maintain a consistent average advance per round under such complex geological conditions demonstrates that the proposed optimization scheme possesses excellent versatility and is not merely effective under specific, isolated conditions. The root cause of this consistent improvement in cyclic advance lies in the high-efficiency burn cut. The large-diameter four-reliever-hole technique ensures the creation of a sufficiently deep and large initial void in every blast, which serves as a critical prerequisite for the success of subsequent shots. Furthermore, the rational arrangement and charging parameters of the auxiliary and perimeter holes ensured full utilization and efficient transmission of the blasting energy.

Based on the visual analysis of the entire excavation and blasting process and the quantitative statistics from long-term production data, the industrial-scale trial was deemed a complete success. The roadway excavation blasting scheme proposed in this study, which was optimized through numerical simulation–particularly featuring the large-diameter four-reliever-hole burn cut and refined smooth blasting parameters–is technologically advanced and practically feasible. Compared to the original method, the new scheme demonstrated significant improvements in increasing cyclic advance, enhancing tunnel contour quality, and reducing damage to the surrounding rock mass. Moreover, the resulting muckpile profile and rock fragmentation were more ideal. The increase in cyclic advance directly accelerates the project schedule, while the improved tunnel contour quality effectively reduces subsequent ground support costs, leading to significant overall economic benefits.

## Discussion

The successful implementation of the optimized blasting scheme, validated through a three-month industrial trial, demonstrates a tangible advancement in drivage practices for large-section drifts in challenging geological environments. This section interprets the key findings in the context of existing literature, acknowledges the inherent limitations of the numerical modeling approach, and outlines the broader implications of this research for the mining industry.

A potential limitation of this study that warrants discussion is the relatively small sample size used for the initial rock mechanics characterization. However, the primary objective of these tests was to provide input for a numerical model aimed at engineering optimization. The subsequent success of the three-month industrial trial, where the optimized blasting scheme performed as predicted, serves as a powerful, engineering-scale validation. This strong correlation between the simulation outcomes and the field results confirms that the laboratory-derived parameters, despite the limited sample set, were sufficiently representative and reliable to guide the practical design and achieve the desired improvements in excavation efficiency and stability.

Our study’s primary quantitative achievements–an average advance per round increase of 6.8% and a half-cast factor consistently exceeding 90%–compare favorably with similar optimization efforts reported in the literature. For instance, Xin et al.^37^ reported a 1.63% improvement in advance per round by optimizing a wedge cut in a similar hard rock environment, highlighting that our 6.8% gain represents a significant and practical improvement. While some studies have achieved higher efficiency gains (around 9%-10%)^38,39^, these were often in less demanding conditions, such as smaller cross-sections or lower in-situ stress fields. The achievement in this study is particularly noteworthy given the large 4.5 m $\times$ 4.0 m cross-section and the high-stress conditions typical of a deep block caving undercut level. Furthermore, the average half-cast factor of over 90% places our results at the upper end of successful smooth blasting applications. While an 80% half-cast factor is widely considered a successful benchmark in hard rock tunneling^40^, the consistent achievement of over 90% in this study signifies exceptional control over blast-induced damage. This superior performance validates the effectiveness of the optimized perimeter hole spacing and decoupled charging, which are crucial for preserving the long-term stability of production infrastructure. Although a detailed financial accounting is beyond the scope of this technical study, the economic feasibility of the optimized scheme can be analyzed through excavation efficiency and quality control. The primary economic benefit stems from the increased advance rate. The field data indicated a stabilized average advance of 3.31 m per round, a 6.8% increase over the original 3.10 m. In the context of a 1,000 m drift development, this improvement eliminates approximately 20 full blasting cycles. Since mining operations incur high fixed costs (labor, ventilation, drainage, and equipment depreciation), reducing the total number of cycles yields significant savings in operational expenditure and accelerates the project timeline. Furthermore, cost reductions are achieved through improved contour quality. The optimized scheme maintained a half-cast factor exceeding 90%, indicating minimal overbreak. This reduction in overbreak has a dual economic advantage: it decreases the volume of waste rock that must be mucked and hauled, and it significantly lowers the consumption of shotcrete and concrete required to backfill over-excavated areas and stabilize rough surfaces. Therefore, the optimized scheme improves the overall ’unit cost per meter’ of drivage, validating its economic efficiency.

A further methodological consideration is the evaluation of blast-induced rock mass disturbance. While blast vibration monitoring is a conventional technique for this purpose, this study prioritized the HCF as the primary key performance indicator. This choice was based on the premise that HCF provides a more direct and integrated measure of the final excavation quality and the degree of damage imparted to the remaining rock mass. Vibration data quantifies the transient energy transfer, whereas the HCF quantifies the ultimate physical result of that energy. As a globally recognized indicator in precision excavation, a high HCF is a direct proxy for the preservation of rock mass integrity at the excavation boundary. Therefore, the consistent achievement of an HCF exceeding 90% during the field trials serves as robust, quantitative evidence that the optimized blasting scheme successfully minimized the disturbance to the surrounding rock, which was the central objective of the research.

It is crucial, however, to acknowledge the limitations of the numerical simulation methodology that guided this optimization. Firstly, the LS-DYNA model, utilizing the RHT constitutive model, treated the andesite rock mass as a homogeneous and continuous medium. This simplification was considered appropriate given the high integrity and high Rock Mass Rating (RMR) of the rock mass at the study site, where the behavior under dynamic loading is predominantly governed by the properties of the intact rock. While this approach does not explicitly account for discontinuities that can influence stress wave propagation, the ultimate validation of this simplification lies in the successful three-month industrial trial. The excellent agreement between the model-driven optimized blasting scheme and the actual field results–specifically achieving a half-cast factor exceeding 90%–strongly suggests that the continuum-based damage model was sufficiently accurate to capture the essential physics required to guide the engineering design. Secondly, while the JWL equation of state accurately models the initial detonation pressure, the simulation does not fully capture the complex, prolonged interaction of high-pressure detonation gases penetrating and extending fractures. This quasi-static gas pressure effect is a primary driver of rock fragmentation, especially between perimeter holes. The damage model in RHT approximates this phenomenon, but a more sophisticated coupled fluid-solid interaction model would be required for a more precise representation. Lastly, the model assumes perfect drilling accuracy in terms of hole location, alignment, and diameter, whereas practical field operations always involve some degree of drilling deviation, which can impact fragmentation and contour control.

It is also important to contextualize the scope of the field validation. The trials were deliberately concentrated at the -450m level over a three-month period, a decision guided by the operational principles of the block caving method. This level represents the critical undercut and production infrastructure, the long-term stability of which governs the success of the entire mining operation. As the vast majority of permanent roadway development is focused at this horizon, it presented the most relevant and high-impact environment for validating our methodology. While the three-month duration does not represent a long-term stability assessment, its purpose was to confirm the efficacy and repeatability of the damage-control blasting methodology during excavation. By successfully controlling the initial excavation-induced damage–the primary precursor to long-term instability–the study provides a validated pathway to ensuring the enduring integrity of this critical mine infrastructure.

Despite these limitations, the broader implications of this work are significant. The study provides a robust and transferable methodology–integrating laboratory testing, calibrated numerical simulation, and rigorous field validation–that can be adapted by other mining operations to solve site-specific blasting challenges. The focus on a large-diameter, four-hole burn cut provides a practical solution that balances performance and cost, offering a valuable alternative to more complex or drill-intensive cut designs. The most significant implication is for mines employing or planning to use the block caving method. The long-term stability of the undercut level is non-negotiable for the success of the entire operation. This research provides a clear technical pathway to minimize excavation-induced damage from the outset, thereby enhancing roadway stability, reducing ground support requirements, and ultimately improving operational safety and economic viability. This proactive approach to damage control is far more effective and economical than reactive ground support rehabilitation measures undertaken after stability issues have already emerged. Future work should focus on three key areas: first, incorporating discrete fracture network (DFN) models to better represent structurally controlled failure; second, conducting long-term geotechnical monitoring of the drifts excavated with the optimized scheme to validate their stability performance over the operational life of the mine; and third, applying this validated methodology to new mining blocks or regions with different geological conditions, recalibrating the model as necessary.Finally, future implementations could benefit from integrating instrumental vibration monitoring to build a comprehensive dataset correlating vibration signatures with the resulting half-cast factors and long-term stability performance.

## Conclusions

This study addresses the dual challenges of ensuring surrounding rock stability and enhancing excavation efficiency during the development of large-section production drifts in mines utilizing the block caving method. This study systematically optimized a drill-and-blast scheme by integrating laboratory rock mechanics testing, LS-DYNA numerical simulations, and full-scale field trials. The optimized approach yielded significant improvements in excavation efficiency. The main conclusions are as follows:

(1) The primary driver for improved excavation efficiency was the optimization of the burn cut parameters. Numerical simulations and field trials demonstrated that the large-diameter four-hole uncharged relief design created a geometrically symmetrical initial cavity. This design effectively balanced the rock breaking volume with the available compensation space, ensuring a consistent cavity formation. Consequently, the average advance per round stabilized at 3.31 m, representing a 6.8% improvement in efficiency compared to the original scheme.

(2) The control of surrounding rock damage was achieved through the refined design of the perimeter holes. Based on the theoretical calculation of crack propagation and stress wave superposition, the perimeter hole spacing was optimized to 600 mm with uncoupled charging. This configuration successfully confined the blast-induced cracks to the excavation boundary. Field results confirmed the effectiveness of this measure, yielding a smooth tunnel profile with an average half-cast factor (HCF) exceeding 90%, significantly reducing overbreak and support requirements.

(3) The industrial implementation at the –450 m level of the JAMA Mine validated the compatibility of these combined measures. The optimized scheme successfully resolved the conflict between rapid advancement and stability control in high-stress environments, providing a robust reference for similar large-section drift excavations in hard rock mines.

## Data Availability

The data used to support the findings of this study are included in the article.
